# Protective effects of *aloe vera* gel (*aloe baberdensis* Miller) on aluminum chloride-induced reproductive toxicity in male Wistar rats

**DOI:** 10.5935/1518-0557.20200082

**Published:** 2021

**Authors:** Busuyi Kolade Akinola, Toluwase Solomon Olawuyi, Victor Okoliko Ukwenya, Lekia Damilola Daniel, Bolanle Christiana Faleye

**Affiliations:** 1 Department of Human Anatomy, Federal University of Technology, Akure. Nigeria; 2 Department of Chemical Science, Joseph Ayo Babalola University, Ikeji-Arakeji. Nigeria

**Keywords:** Testis, aloe vera gel, aluminum chloride, semen

## Abstract

**Objective:**

This study aims at investigating the protective effects of aloe vera gel on aluminum chloride-induced testicular toxicity of adult Wistar rats.

**Methods:**

Twenty male Wistar rats were randomly divided into four groups (A, B, C, and D) with five animals per group. Group A serves as the control group and received distilled water (1ml/Kg). Group B received distilled water (1ml/Kg) with 100mg/kg b.wt of aluminum chloride daily. Group C received 100mg/kg b.wt of aluminum chloride with 600mg/kg b.wt of Aloe vera gel daily. Group D received 100mg/kg b.wt of aluminum chloride with 5mg/kg b.wt of vitamin C daily. The animals were fed on standard laboratory animal diet and water *ad libitum*. Administration was via oral cannula for four weeks. The rats were slaughtered using cervical dislocation. The testes were harvested for seminal, biochemical and histological analysis.

**Results:**

The results demonstrate that the administration of aloe vera gel (AVG) is capable of preventing testicular toxicity due to aluminum chloride. Aluminum chloride caused a significant change in the testes and seminal parameters of group B when compared to the control animals. The level of Nitric oxide (NO) increased and the level of Superoxide dismutase (SOD) decreased significantly in rats treated with aluminum chloride.

**Conclusions:**

The administration of aloe vera gel showed a preventive response in aluminum-induced testicular toxicity of rats as evidenced by histological and biochemical analysis.

## INTRODUCTION

Male infertility caused by exposure to heavy metals is a global problem [Wong and Cheng, 2011], and it accounts for 45% of infertility cases (Kumar & Singh, 2015). Testicular toxicity, an injury to the testis, is associated with impaired testicular function caused by free radicals derived from oxygen on exposure to drugs, chemicals and non-infectious agents (Liu *et al*., 2017). A report has confirmed that oxidative stress is the chief cause of testicular toxicity (Gupta & Verma, 2018).

Aluminum is a trivalent cation found in its ionic form in most animals, plants and in natural waters everywhere (Jiang *et al*., 2008). Aluminum enters the human body via food, water, drugs, air, aluminum ware and containers, as well as in many manufactured food stuffs, such as processed cheese, baking powders, cake mixes, pancake mixes, frozen dough (Nayak, 2002). Moreover, high concentrations of aluminum in spermatozoa and seminal plasma of humans decreases sperm viability and motility (Yousef *et al*., 2007). Guo *et al*. (2002) described the oxidative damage and testicular toxicity caused by aluminum to the reduction in testis acetylcholinesterase (AChE) activity. Testicular aluminum buildup, necrosis of spermatocytes/spermatids, and a significant decline in fertility were also detected in both male rats and mice (Guo *et al*., 2005a, Sharma *et al*., 2003). Aluminum may cause male reproductive toxicity through different mechanisms such as inducing oxidative stress, interfering with spermatogenesis and steroidogenesis, damaging cell signaling, disrupting the blood-testis barrier, and affecting the endocrine system (Pandey & Jain, 2013). Aluminum has also been stated to cause testicular toxicity by increasing nitric oxide level (Guo *et al*., 2005b). Another study showed that aluminum reduces antioxidants and increases lipid peroxidation (Yousef & Salama, 2009).

*Humankind has used aloe vera* for thousands of years in folk medicine for therapeutic properties, especially on the skin. This plant is one of the oldest known medicinal plants, and its first documented use by humans dates back to an Egyptian papyrus from 3,500 BC (Crosswhite & Crosswhite, 1984). Moreover, modern clinical use began in the 1930s with reports of successful treatment of x-ray and radium burns (Surjushe *et al*., 2008). The leaves are composed of three layers: an inner gel, a yellow sap and the outer thick layer of 15-20 cells called ‘rind’ (Eshun & He, 2004). When the whole leaf of *Aloe vera* is used, it is difficult to distinguish whether its biological effects are attributed to the gel or the latex because, during the gel preparation, exudates compounds may infiltrate (Reynolds & Dweck, 1999). The phytochemical analysis of *Aloe vera* gel shows that it has the following compounds; polysaccharides, steroids, organic acids, antibiotic agents, amino acids and minerals, which has skin soothing and cells protecting effects (Hamman, 2008). Different studies reported the hypoglycemic, hypotensive, liver protective and blood purifying properties of Aloe vera (Hamman 2008; Nwajo, 2006). This study investigates the protective effects of *Aloe vera* leaf gel (*aloe baberdensis* Miller) on aluminum chloride-induced testicular toxicity of male Wistar rats.

## MATERIALS AND METHODS

### Experimental animals

We used twenty male Wistar rats weighing between 120 - 140g for this study. The rats were obtained from the Central animal facility of the Biochemistry Department, Federal University of Technology Akure, Ondo State, Nigeria. The rats were kept in ventilated cages at optimum temperature and 12 hours light/dark cycles. The animals were fed on standard laboratory animal diet and water *ad libitum*. The experiment was carried out in accordance with current rules and guidelines that have been established for the care of laboratory animals (National Research Council, 2011). The rats were acclimatized for two weeks before the commencement of the experiment.

### Preparation of *aloe barbadensis* leaf gel

Healthy and mature *Aloe vera* leaves were harvested from the Botanical garden of the Federal University of Technology, Akure, Ondo State, Nigeria. The leaves were washed with clean water, the thick epidermis was peeled off and the aloe vera gel was removed carefully. The gel was then homogenized with an electric blender. The homogenate was concentrated by filtration using whattman’s filter paper size 2. The thickened concentrated gel and the filtrate were kept at 4^o^C until used.

### Experimental design

The 20 male Wistar rats were randomly divided into 4 groups (A, B, C, and D) with five animals per group.

**Group A** served as the control group and received oral administration of distilled water (1ml/Kg b.wt) daily for 4 weeks.

**Group B** received oral administration of distilled water (1ml/Kg b.wt) with 100mg/kg b.wt of aluminum chloride daily for 4 weeks.

**Group C** received oral administration of 100mg/kg b.wt of aluminum chloride with 600mg/kg b.wt of *Aloe vera* gel daily for 4 weeks.

**Group D** received oral administration of 100mg/kg b.wt of aluminum chloride with 5mg/kg b.wt of vitamin C daily for 4 weeks.

At the end of the experiment, the animals were slaughtered using cervical dislocation and the testes removed for histological analysis.

### Histological procedures

The testes were excised and immediately fixed in bouin’s fluid, and thereafter processed routinely for paraffin embedding; Micro-sections of 5µm were obtained with a rotary microtome and processed with Hematoxylin and Eosin stain (H&E). We examined the stained slides under a microscope using different magnifications (X40 and X100) and took photomicrographs.

### Semen Analysis

The cauda epididymis was removed, incised and semen prepared for analysis. Sperm count was done according to the method reported by Ekaluo *et al.* (2008). The sperm viability test was determined using the Eosin-Nigrosin staining technique (Bjorndahl *et al*., 2003). Sperm head abnormality test was done according to Ekaluo *et al*. (2009). Sperm motility and pH were done as reported by Ekaluo *et al*. (2008).

### Biochemical analysis

The testes were excised and homogenized in 0.1mmol/L Tris buffer (pH 7.4) and used for the biochemical estimations. From the left testis, homogenates were subjected to a biochemical assay to estimate the superoxide dismutase (SOD) activity by the method described by Misra & Fridovich (1972). Nitric oxide (NO) in the tissue was assayed according to the method by Moshage *et al.* (1995). This was done to evaluate the effect of AVG on oxidative stress markers and to validate the claim that aluminum chloride causes testicular toxicity by inducing oxidative stress.

### Statistical analysis

Using Graph pad prism (version 8.0.), the data obtained was analyzed and expressed as mean ± S.E.M (standard error of mean) and subjected to one-way analysis of variance (ANOVA). This was then subjected to a posthoc test using the student Neumann Keul's method and values were considered statistically significant if *p*<0.05.

## RESULTS

### Histological sections of testes across the group

**Plate 1:**
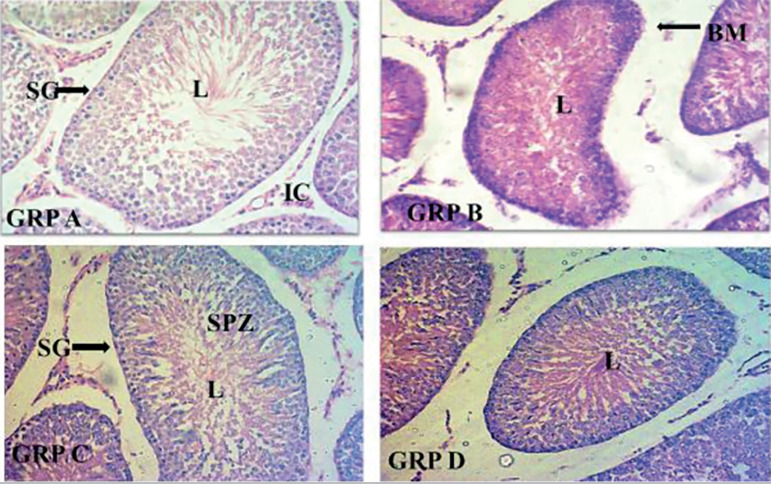
Photomicrograph of testes showing: Group A with normal histological architecture characterized by typically organized layers of spermatogenic cells; Group B with distorted tubular architecture and disorganization of the spermatogenic cells in seminiferous tubules and major pathological changes in the lumen (L); Group C with restored microarchitecture of the testicular morphology, distribution of epithelial lining, partially restored lumen (L); Group D with restored microarchitecture of the testicular morphology, distribution of epithelial lining, partially restored lumen (L). Stains: Hematoxylin and Eosin (H&E). Magnification X100.

**Plate 2:**
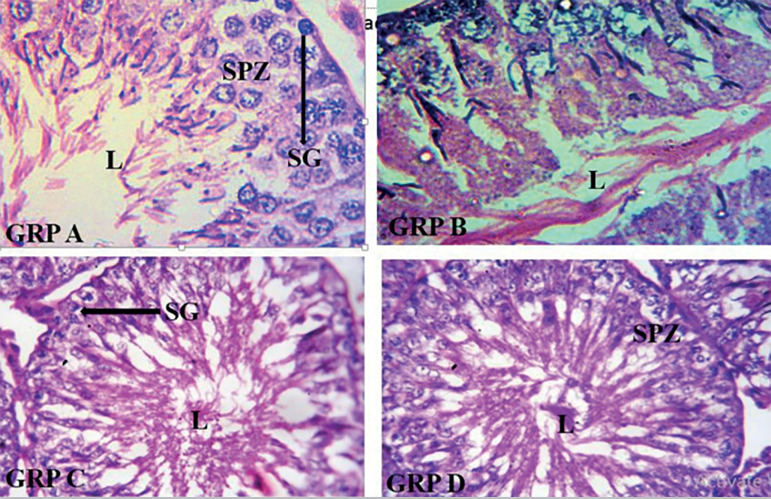
Photomicrograph of testes histological architecture showing: Group A with normal histological architecture characterized by typically organized layers of spermatogenic cells; Group B with distorted tubular architecture and disorganization of the spermatogenic cells in seminiferous tubules, major pathological changes in the lumen (L); Group C with restored microarchitecture of the testicular morphology, distribution of epithelial lining, partially restored lumen (L); Group D with restored microarchitecture of the testicular morphology, distribution of epithelial lining, partially restored lumen (L). Stains: Hematoxylin and Eosin (H&E). Magnification X400.

Plates 1 and 2 show the results. The photomicrograph of the Group C’s histological section revealed that the administration of Aloe Vera gel (AVG) ameliorates the cytoarchitecture and histomorphological distortions evident in group B (induced with AlCl_3_). The observations of the control group (A), showed normal testicular morphology. This is in complete contrast to group B, which showed a structural distortion typical of testicular toxicity. Group D also shows that Vitamin C has a similar effect on AlCl_3_-induced testicular toxicity when compared with the group treated with AVG.

### Superoxide dismutase (SOD)

**Figure 1:**
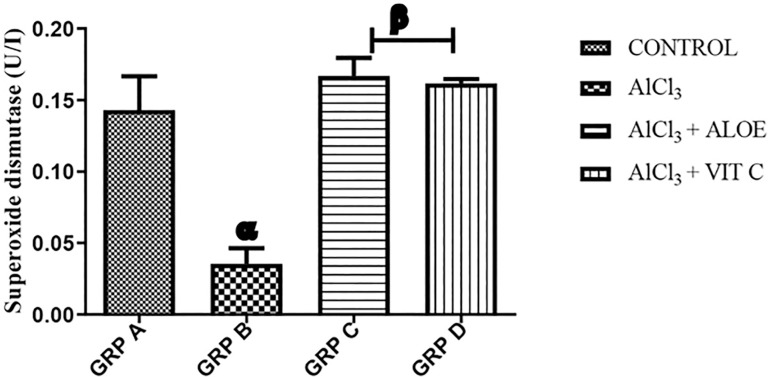
Graph showing serum SOD activity across the groups. Each value represents Mean±SEM, n=5 readings. Value of *p*<0.05 was considered significant. The values with superscript α=significantly lower from group A, β=significantly higher from group B. SOD - Superoxide Dismutase.

Figure 1 illustrates the effects of Aloe vera gel on SOD activity in the serum of Wistar rats with Aluminum chlorideinduced testicular toxicity. Group A (control) presented an average concentration of SOD of 0.14±0.04U/L. Group B presented an average concentration of SOD of 0.04±0.02U/L. Group C presented an average SOD concentration of 0.17±0.02U/L while group D presented an average concentration of 0.16±0.01U/L. There was a significant decrease (*p*<0.001) in the serum level of SOD in group B rats when compared with the normal controls (group A). The serum level of SOD is also significantly different when comparing the treated groups with the non-treated group (group B) (*p*<0.005). There was no significant difference between the treated groups when compared to each other.

### Nitric oxide (NO)

**Figure 2:**
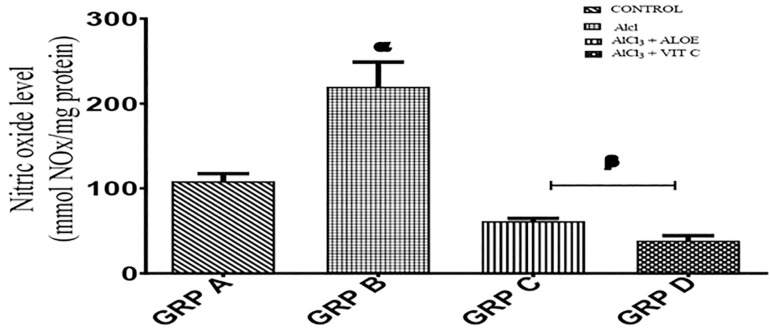
Graph showing serum NO activity across the groups. Each value represents Mean+SEM, n=5 readings. Value of *p*<0.05 was considered significant. The values with superscript α=significantly differs from group A, β=significantly different from group B. NO - Nitric Oxide.

Figure 2 illustrates the effects of Aloe vera gel on the Nitric oxide (NO) activity in the serum of Wistar rats with Aluminum chloride-induced testicular toxicity. Group A (control) presented an average concentration of NO of 108.4±9.0mmol/mg protein. Group B presented an average concentration of NO of 219.7±29.2mmol/mg protein. Group C presented an average concentration of NO of 61.4±3.3mmol/mg protein; while group D presented an average concentration of 38.4±5.9 mmol/mg protein. There was a significant increase (*p*<0.005) in the serum level of NO in group B rats when compared with the normal controls (group A). The serum level of NO is also significantly different when comparing the treated groups with the non-treated group (group B) (*p*<0.001).

### Semen analysis

#### Sperm volume

**Figure 3:**
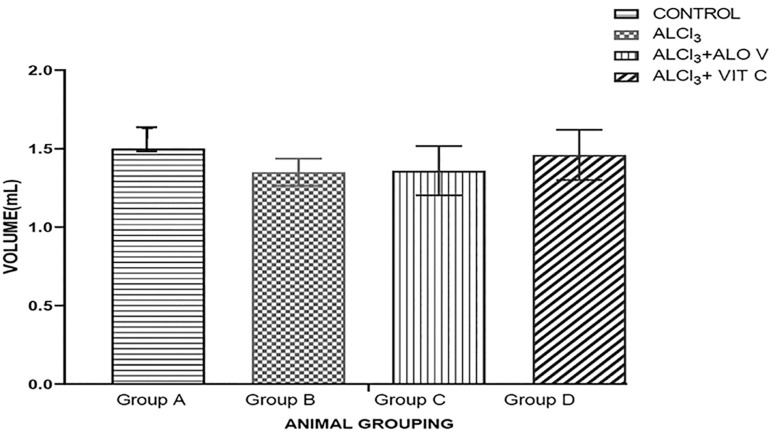
Graph showing sperm volume across the groups. Bars represent Mean ± SEM. p<0.05. n=5. Each value represents Mean±SEM, n=5 readings. Value of p<0.05 was considered significant.

There was an increase in sperm volume among the rats in the group treated with *Aloe vera* gel (group C) when compared with the control group. Group B shows a decrease in the sperm volume when compared with the control group (Figure 3).

#### Sperm motile count

**Figure 4:**
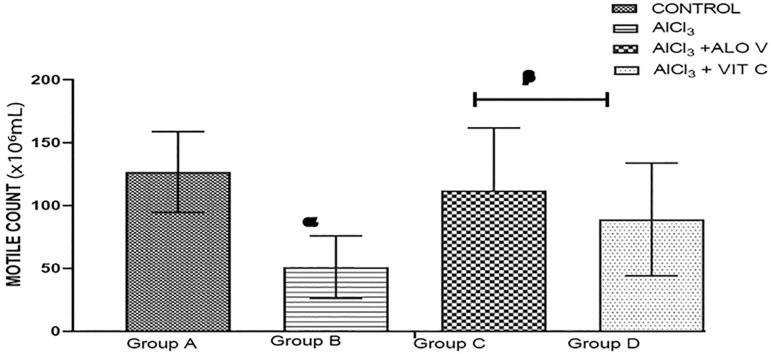
Graph showing the motile count of sperm cells across groups. Each value represents Mean+SEM, Value of *p*<0.05 was considered significant. Charts with superscript α (alpha) is significantly different from group A, superscript β (beta) is significantly different from group B.

This increased significantly (*p*<0.05) in the group treated with Aloe Vera gel and vitamin C (group C and D, respectively) when compared with group B. The group induced with AlCl_3_ shows a decrease in sperm motile count when compared with other groups (Figure 4).

#### Total sperm count

**Figure 5:**
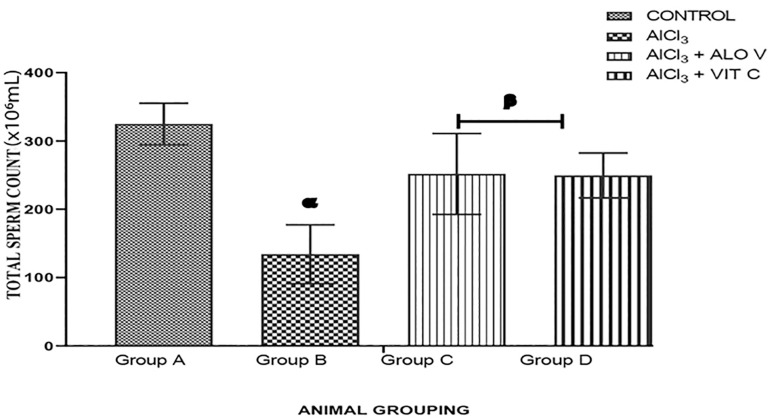
Graph showing the total sperm count across the groups. Each value represents Mean±SEM, n=5 readings. A *p* value <0.05 was considered significant. The values with superscript α are significantly different from group A, β=significantly different from group B.

Group A (control) presented an average Total Sperm Count (TSC) of 325.0±52.1x10_6_mL. Group B presented an average TSC of 171.0±55.1x10_6_mL. Group C presented an average TSC of 333.0±73.7 x10_6_mL while group D presented an average TSC of 282.0±14.7x10_6_mL. There was a significant reduction (*p*<0.005) in the group treated with AlCl_3_ (group B) when compared with the control group. Groups treated with AVG and vitamin C (group C and D) shows a significant increase (*p*<0.005) when compared with group B (Figure 5).

### Sperm concentration count

**Figure 6:**
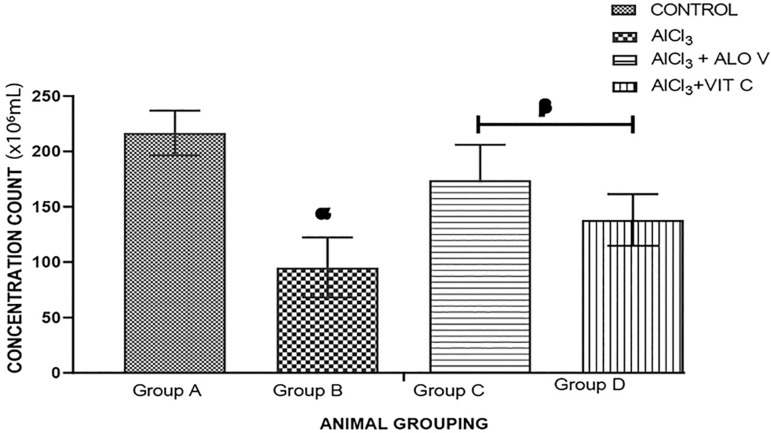
Graph showing sperm concentration count across the groups. Each value represents Mean±SEM, n=4 readings. A *p* value <0.05 was considered significant. The values with superscript α are significantly different from group A, β=significantly different from group B.

Group A (control) presented an average Sperm Concentration Count (SCC) of 216.7±35.1x10_6_mL. Group B presented an average SCC of 120.0±26.5x10_6_mL. Group C presented an average SCC of 205.0±20.8x10_6_mL while group D presented an average SCC of 157.5±33.0x10_6_mL. Sperm concentration significantly reduced in the group treated with AlCl_3_ (group B) when compared with the control group. Groups treated with Aloe Vera gel and vitamin C (groups C and D) showed a significant increase (*p*<0.005) when compared with group B (Figure 6).

### Sperm morphology

**Figure 7:**
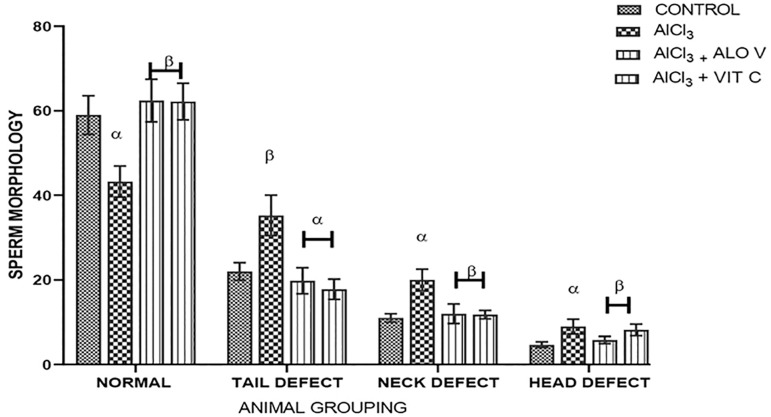
Graph shows the sperm morphology across the groups. Each value represents Mean±SEM, n = 5 readings. A *p* value <0.05 was considered significant. Effects of Aloe vera gel on sperm morphology. The values with superscript α are significant different from Group A, β=different from Group B

There was a significant difference (*p*<0.05) in sperm morphology in the treated groups (C & D) when compared to the group induced with AlCl_3_. The morphology recovery also appears to be treatment-dependent as group C was found to be significantly different from group D (Figure 7).

### Sperm progressive assessment

**Figure 8:**
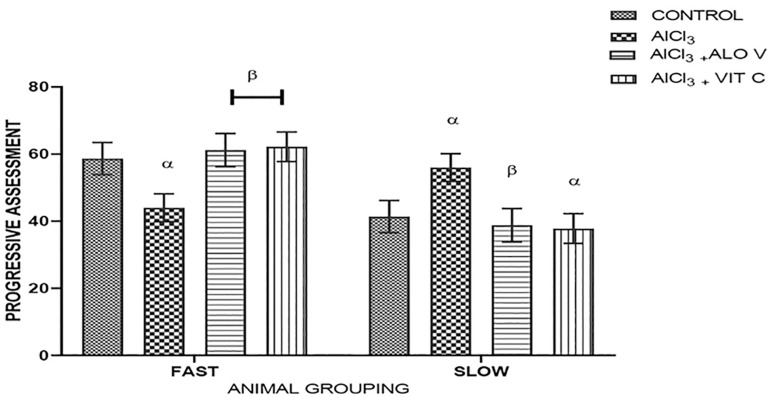
Graph showing the progressive assessment of the sperms across the groups. Each value represents Mean±SEM, n=5 readings, a *p* value <0.05 was considered significant. The values with superscript α are significantly different from Group A, β=different from Group B.

There were higher fast movements in the group treated with Aloe Vera gel (group C), followed by group D treated with vitamin C. Slow movement was remarkably noticeable in group B, induced with AlCl_3_ (Figure 8).

## DISCUSSION

Heavy metals are considered dangerous substances causing health hazards to humans and animals through progressive irreversible accumulation in their bodies, because of repeated consumption of small amounts of these metals. Aluminum chloride is capable of damaging the organism in many ways, due to its high affinity to various tissues and its tendency to accumulate (Aitken & Roman, 2008).

The present study revealed that the administration of Aloe vera gel protects against the damaging effects of AlCl_3_ on the histomorphological features of the testes. It was clearly observed that the group treated with AVG showed near normal testicular morphology and compares fairly well with Vitamin C, a known antioxidant. This is clearly demonstrated by the reversal of distorted histological architecture seen in the photomicrograph of AVG treated rats. This is in agreement with the findings in similar research, which reported that Aloe vera was effective in ameliorating histopathological changes in the testes following bisphenol A induced testicular toxicity (Behmanesh *et al*., 2018).

Our findings in this study showed that aluminum chloride has negative effects on sperm motility, sperm count, viability, morphology and it increases the percentage of abnormal sperms. This finding is in support of past studies that aluminum chloride has the ability to reduce reproductive capacity (Guo *et al.,* 2005b; Buraimoh *et al*., 2012). In the current study, results revealed that the accumulation of aluminum chloride in the body could be a predisposing factor to male infertility, as recorded in the semen parameters and sperm morphology. This is because the accumulation of aluminum chloride in the body can lead to elevated concentrations of aluminum chloride in the testes (Guo *et al.,* 2002). In this study, we also observed that aloe vera gel attenuated the detrimental effects of aluminum chloride on the semen parameters. Therefore, it is plausible to suggest that the effects of aloe vera gel on sperm volume, sperm motility, sperm counts and morphology could be partly mediated by its counteraction on oxidative stress within rat reproductive organs, via its antioxidant properties (Ikeno *et al.,* 2002). The aluminum chloride administration with vitamin C treatment also attenuated testicular damage induced by aluminum chloride treatment, as shown by the normal sperm count, normal sperm morphology and low histopathological changes in comparison with the group that received aluminum chloride only (group B). The protective effect of vitamin C is accompanied by normalization of antioxidant activity in testis (Muthu & Krishnamoorthy, 2012). However, aloe vera gel demonstrates similar antioxidant properties when compared to vitamin C.

In agreement with the report from Yousef & Salama (2009), the results of this study showed that aluminum reduced superoxide dismutase (SOD) activity. SOD protects spermatozoa against spontaneous oxygen toxicity and lipid peroxidation. Since Reactive Oxygen Species (ROS) have been indicated to have a role in steroidogenesis and gametogenesis, the mentioned effects might have been responsible for the poor sperm quality seen in aluminum-treated rats. Aluminum can incite the oxidation of molecules in the body, thereby resulting in oxidative stress. Oxidative stress has been shown to play an important role in causing male infertility by inducing defects in sperm functions. Reactive oxygen species (ROS) are central to a host of pathologies, including inflammation, toxicity, and endocrine disruption by environmental chemicals, and are degraded by the organized system of antioxidants. ROS damages almost all macromolecules of the cell, causing impairment of cellular functions (Arumugam *et al*., 2014). Meanwhile, Aloe vera exhibits protective effects against oxidative damage by decreasing the levels of free radicals, through its free radical scavenging activity, particularly against oxygen radicals. Oxidative stress through the generation of ROS had been proposed as one of the possible mechanisms of aluminum chloride toxicity on male reproductive functions. It has been proven that aluminum chloride increased the production of ROS by increasing the generation of testicular hydrogen peroxide and hydroxyl radicals in experimental rats (Exley, 2004; Yousef & Salama, 2009). Vitamin C treatment in this study prevents testicular damage and enhances sperm quality. Accumulating evidence suggests that the protective effect of vitamin C against oxidative damage is due to its anti-oxidative properties (Wang *et al.,* 2002). However, aloe vera gel improved the sperm quality by scavenging the ROS and enhanced testicular enzymatic antioxidant ability. The result of this study also shows that aluminum chloride significantly increased nitric oxide activity compared to the control and treated groups (groups A, C, and D) affirming the fact that it causes damage by inducing oxidative stress.

## CONCLUSION

Based on the present study, we can conclude that aluminum chloride distorted testicular histo-architecture *via* altered reproductive biochemistry. The toxic effects induced by aluminum chloride were ameliorated after treatment with aloe vera gel. Aloe vera gel showed similar anti-oxidative properties when compared with ascorbic acid, a known anti-oxidant.
